# Impaired Expression of Prostaglandin E_2_ (PGE_2_) Synthesis and Degradation Enzymes during Differentiation of Immortalized Urothelial Cells from Patients with Interstitial Cystitis/Painful Bladder Syndrome

**DOI:** 10.1371/journal.pone.0129466

**Published:** 2015-06-09

**Authors:** John O. Marentette, Robert E. Hurst, Jane McHowat

**Affiliations:** 1 Department of Pathology, Saint Louis University School of Medicine, 1402 S. Grand Blvd., St. Louis, MO 63104, United States of America; 2 Department of Urology, Oklahoma University Health Sciences Center, 940 S. L. Young Blvd., Oklahoma City, OK, 73104, United States of America; National Cancer Institute at Frederick, UNITED STATES

## Abstract

**Purpose:**

The differentiated superficial cells of the urothelium restrict urine flow into the bladder wall. We have demonstrated that urothelial cells isolated from bladders of patients with interstitial cystitis/painful bladder syndrome (IC/PBS) fail to release PGE_2_ in response to tryptase. This study examines the expression of PGE_2_ synthesis and degradation enzymes in urothelial cells during differentiation.

**Materials and Methods:**

We measured immunoprotein expression of cyclooxygenase-2 (COX-2), prostaglandin E_2_ synthase (PGES) and 15-hydroxyprostaglandin dehydrogenase (PGDH) in human urothelial cells and in immortalized urothelial cells isolated from the bladders of IC/PBS patients or normal subjects during stratification and differentiation produced by increased calcium and fetal bovine serum (Ca/FBS) in the culture medium for 1, 3 and 7 days.

**Results:**

PGES immunoprotein expression increased during differentiation in normal and IC/PBS urothelial cells. COX-2 expression also increased in cells from normal patients following differentiation. Remarkably, no COX-2 expression was detectable in urothelial cells isolated from 3 out of 4 IC/PBS patients. PGDH immunoprotein expression decreased in normal cells after 1 and 3 days of Ca/FBS addition, but returned to normal after 7 days. PGDH expression was unchanged during differentiation at 1 and 3 days, but was more than 2-fold higher at 7 days compared to day 0 in the IC/PBS cells. Urothelial cells isolated from IC/PBS patients demonstrated no PGE_2_ release in response to tryptase under any of the experimental conditions studied.

**Conclusions:**

Taken together, our results indicate that PGE_2_ release is compromised during stratification and differentiation in IC/PBS urothelium and may contribute to impaired barrier function.

## Introduction

Interstitial cystitis/painful bladder syndrome (IC/PBS) is associated with increased activated mast cell numbers in the bladder [1–3], impairment of the barrier function [4–6] of the urothelium and neurogenic inflammation [7–9]. Mast cells are the primary effectors of type 1 IgE-mediated immune reaction and release a battery of stored and newly formed mediators upon activation, including biogenic amines such as histamine, neutral proteases such as tryptase, chymase, cathepsin and carboxypeptidase, arachidonic acid metabolites, chemokines and cytokines. Bladder biopsies from patients diagnosed with IC/PBS have demonstrated an increase in the number of IL-6 positive mast cells, and mast cell mediators such as tryptase and histamine are detected in the urine of IC/PBS patients.

We have demonstrated previously that tryptase stimulation of human urothelial cells results in activation of calcium-independent phospholipase A_2_ (iPLA_2_) and increased release of prostaglandin E_2_ (PGE_2_) [10]. PGE_2_ is widely accepted to be cytoprotective in the epithelium and is involved in wound repair and cell motility. Since IC/PBS is associated with impairment of urothelial integrity, it is compelling to suggest that PGE_2_ may be beneficial in this disease.

Following activation of phospholipase A_2_, arachidonic acid is hydrolyzed from the *sn*-2 position of membrane phospholipids. Free arachidonic acid is hydrolyzed by COX-2 to prostaglandin H_2_ (PGH_2_), the precursor for prostaglandin synthesis [11]. Prostaglandin E synthase (PGES) synthesizes PGE_2_ from PGH_2_. The COX-2/PGE_2_ pathway has been well studied in regards to chronic inflammation and cancer development [12, 13]. Bioinformatics analysis of urothelial cells treated with antiproliferative factor (APF), has implicated COX-2 as being involved in IC/BPS however, the extent is unknown [14]. As IC/BPS is considered a chronic inflammatory disease, it seems compelling to determine the role of this pathway in urothelial cell cultures. In a previous study, we demonstrated the absence of PGE_2_ release from immortalized cells isolated from IC/PBS bladders incubated with tryptase, and we proposed that this absence may contribute to impaired urothelial cell barrier function and repair in these patients. We determined that the absence of PGE_2_ release may be a result of increased 15-hydroxyprostaglandin dehydrogenase (PGDH) and increased PGE_2_ degradation in urothelial cells from IC/PBS bladders.

The urothelium is composed of stratified transitional epithelium of 3 cell types: basal, intermediate, and superficial. It has been proposed that urothelial cells acquire their mature characteristics through differentiation. The most differentiated, superficial cells develop tight junctions composed of proteins, such as occludin, on adjacent cells that restrict urine movement and abnormalities in barrier function may be involved in syndromes such as IC/PBS. Recently, PGDH has been implicated in maintaining urothelial differentiation. In this study, we measured protein expression of PGDH, cyclooxygenase-2 (COX-2) and prostaglandin E_2_ synthase (PGES) in primary cultures of human urothelial cells (HUC) and immortalized urothelial cells isolated from the bladders of IC/PBS and normal patients during differentiation produced by increased calcium and fetal bovine serum (FBS) in the culture medium for 1, 3 and 7 days.

## Materials and Methods

### Cell Culture

Normal human urothelial cells (HUC) were obtained from ScienCell Research Laboratories (Carlsbad, CA), cell isolations from 3 separate donors were used. Urothelial cells isolated from normal bladder (5 separate donors) and the bladder of patients with IC/PBS (5 separate donors) were immortalized with HPV type 16E6E7as described previously [15, 16]. Samples were obtained from IC/PBS patients by biopsy or bladder washing during cystoscopy. Samples were collected according to an Oklahoma University Health Sciences Center Institutional Review Board approved protocol following informed written consent.

Urothelial cells were grown in EpiLife Media (Cascade Biologics, Inc. Portland, OR) with calcium (0.06mM), growth factor supplements provided by the manufacturer and penicillin (20U/ml)/streptomycin (100mg/ml) (Sigma Chemical Company, St. Louis, MO). After reaching confluence, cells were stratified in the same medium with 10% fetal bovine serum (FBS) and additional 1.0 mM calcium (Ca/FBS). Experiments were conducted at 1, 3, and 7 days after Ca/ FBS addition. These cells in culture show expression of adherens junctions, tight junctions and claudins [17].

### Microscopy

Cultured urothelial cells grown on Transwell inserts were fixed with 3.5% glutaraldehyde in 0.1 M sodium cacodylate buffer (pH 7.25), 5%sucrose, 2 mM calcium chloride for 3 hours and then for 16 hours in fresh fixative at 4°C. Cells were washed in 0.1 M sodium cacodylate buffer, 5% sucrose at room temperature and postfixed in 1% osmium tetroxide in 0.1 M sodium cacodylate buffer, 5% sucrose for 3 hours. Cells were washed in water, incubated 1 hour in 2% aqueous uranyl acetate, dehydrated through graded ethanols to 100%, rinsed in propylene oxide, and infiltrated with a 1:1 mixture of Polybed resin (Polysciences, Inc., Warrington, PA) and propylene oxide for 3 hours. Cells were incubated in Polybed resin for 3 hours, transferred to fresh resin, and polymerized overnight at 70°C. Small plastic blocks containing selected cell areas were cut, attached to blank plastic stubs, trimmed and sectioned using a Reichert Ultracut E ultramicrotome (Depew, NY). “Thick” (0.5 μm) sections were cut, heat attached to glass slides, stained with toluidine blue and examined with a light microscope.

### Immunoblot Analysis

Urothelial cells were suspended in ice-cold buffer containing: (mM) HEPES 20, sucrose 250, dithiothreitol 2, EDTA 2, EGTA 2, β-glycerophosphate 10, sodium orthovanadate 1, phenylmethylsulfonyl fluoride 2, leupeptin 20 μg/ml, aprotinin 10 μg/ml and pepstatin A 5 μg/ml (pH 7.6), and sonicated on ice for 3 bursts of 6 sec. The sonicate was centrifuged at 20,000xg at 4°C for 20 min to remove cellular debris and nuclei. Cytosolic protein was mixed with SDS-PAGE sample buffer, heated at 95°C for 5 min and loaded onto 10% polyacrylamide gels. Proteins were separated by electrophoresis under reducing conditions (SDS-PAGE) and transferred to PVDF membranes. Non-specific sites were blocked with 0.05% Tween 20 and 5% non-fat milk overnight and then incubated with appropriate primary and horse-radish peroxidase (HRP)-linked secondary antibodies. After washing, proteins were detected by the enhanced chemiluminescence method (Supersignal Ultra, Pierce Chemical) and quantified by densitometric analysis (Bio-Rad). The density of bands of interest were normalized to actin content for each sample.

### Measurement of PGE_2_ Release

Human urothelial cells and immortalized urothelial cells from normal and IC/PBS urothelial cells were grown to confluence in 16-mm tissue culture dishes. Cell were washed twice with Hanks balanced salt solution (HBSS) containing (in mmol/L) 135 NaCl, 0.8 MgSO_4_, 10 HEPES (pH 7.6), 1.2 CaCl_2_, 5.4 KCl, 0.4 KH_2_PO_4_, 0.3 Na_2_PO_4_, and 6.6 glucose. After being washed, 0.5 mL of HBSS with 0.36% bovine serum albumin was added to each culture well. Cells were then stimulated with tryptase (20ng/mL) and PGE_2_ release was measured using an immunoassay kit (R&D Systems).

### Statistical Analysis

Statistical analysis was performed using Students’ t test or ANOVA followed by Dunnett’s test. The difference between groups was considered significant at a level of p<0.05. All assays were performed at least four times.

## Results

**Fig 1:**
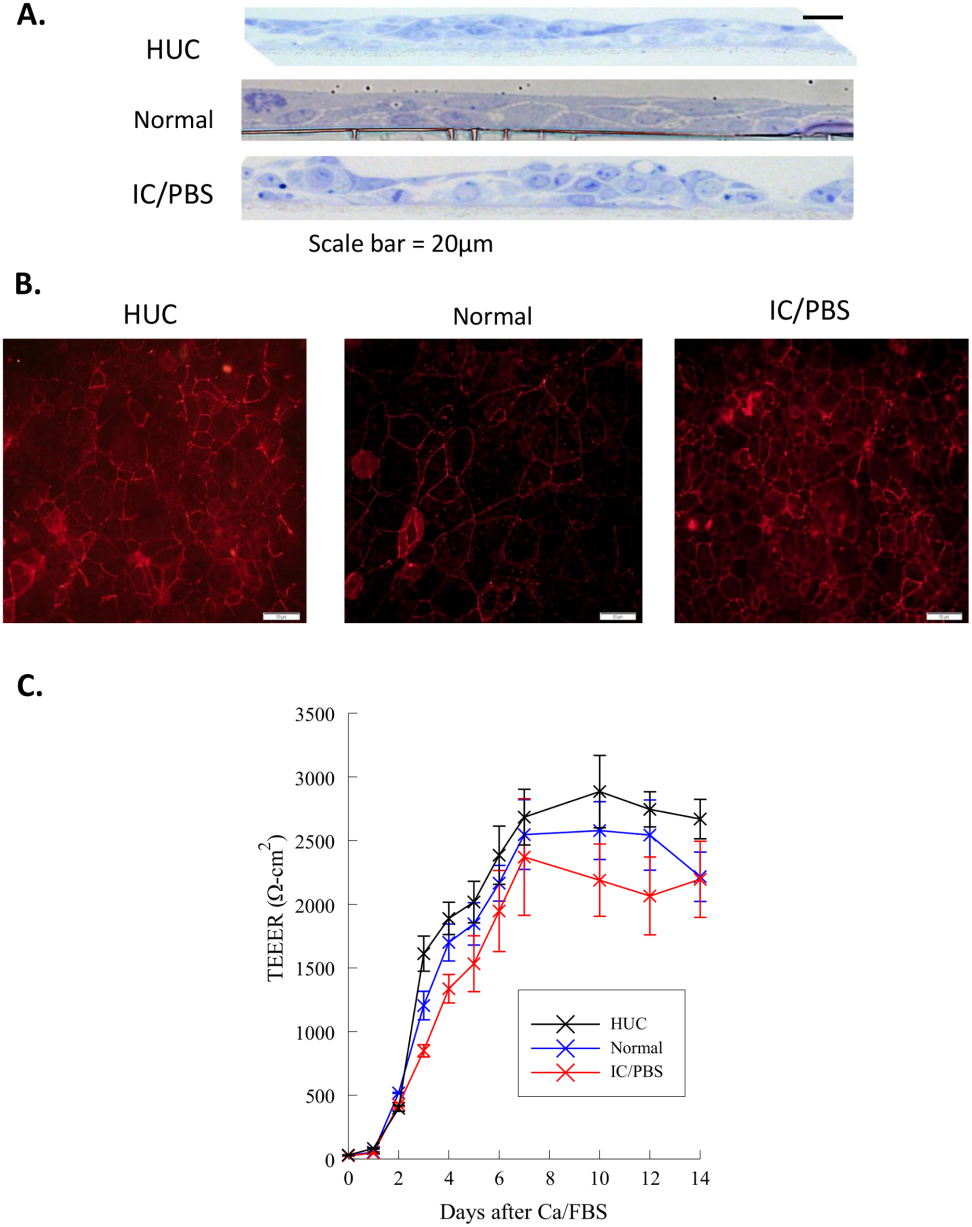
Urothelial cells differentiate into a stratified epithelial culture consisting of thin, tightly apposed apical superficial cells and more loosely connected underlying cells. (A) Light micrographs were taken of cultured human urothelial cells (HUC), immortalized urothelial cells from a normal patient (Normal) or an IC/PBS patient (IC/BPS) that had been stained with toluidine blue. (B) Occludin expression in stratified urothelial cells after 3 days of Ca/FBS addition. (C) Development of trans epithelial electrical resistance (TEER) over 14 days.

Primary urothelial cells and immortalized cells differentiate into a stratified epithelial culture with thin, tightly opposed apical superficial cells and more loosely connected underlying cells after 3 days of additional calcium and FBS incubation (Fig 1A). Urothelial cells showed expression of occludin, indicative of a fully differentiated urothelial cell, following 3 days of Ca/FBS addition (Fig 1B) and fully developed transepithelial electrical resistance (TEER) after 7 days of Ca/FBS addition (Fig 1C). This figure highlights that the three cell types used for the following experiments represent cell cultures of fully differentiated, stratified urothelial cells and that immortalization of the normal and IC/BPS derived urothelial cells did not alter their physical properties. This is validated by comparison to the non-immortalized primary human urothelial cell (HUC) cultures.

**Fig 2:**
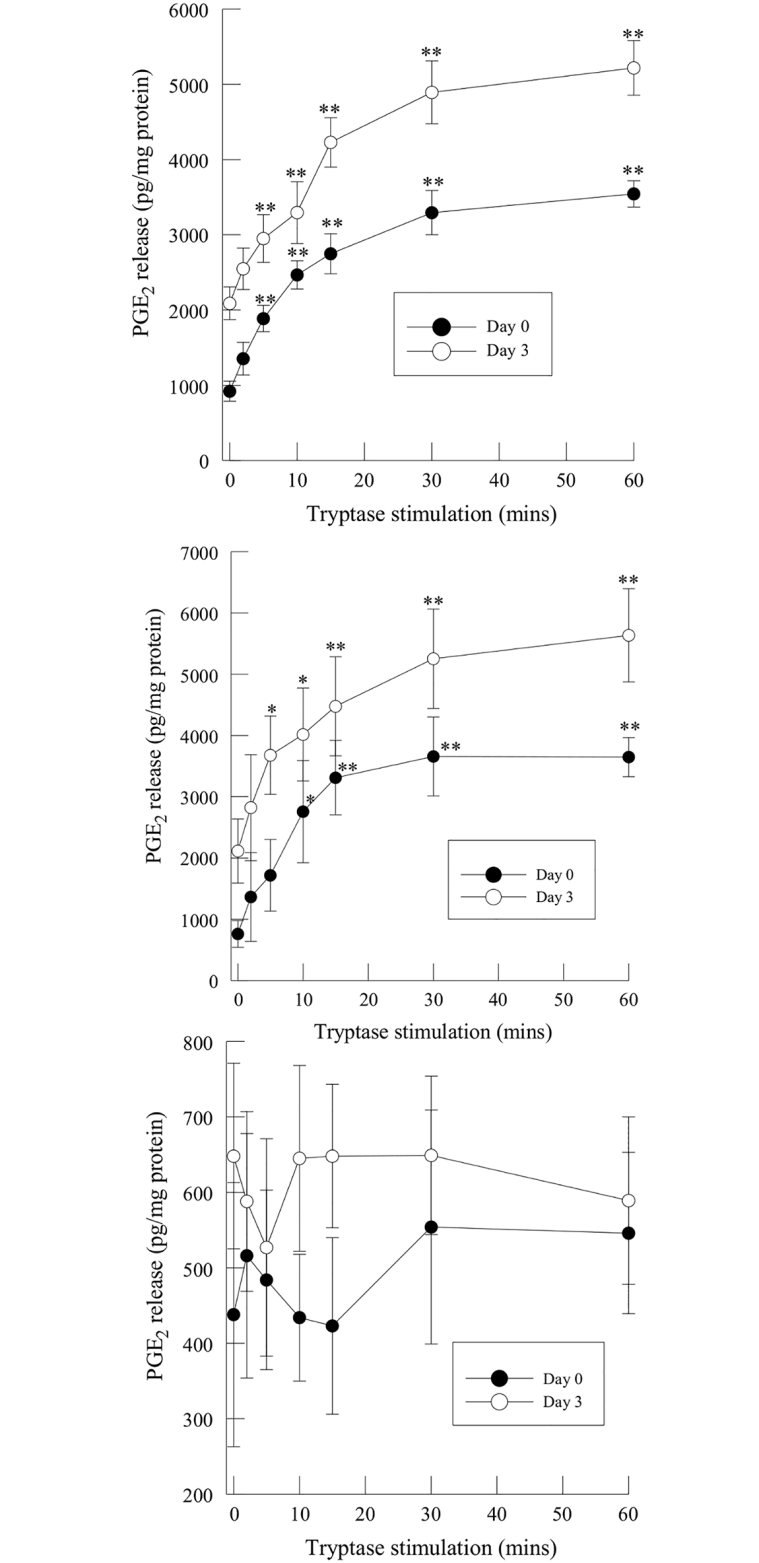
PGE_2_ release in immortalized urothelial cells isolated from normal subjects (upper panel), primary human urothelial cells (HUC, middle panel) and IC/PBS patients (lower panel) after 20 ng/ml tryptase stimulation. PGE_2_ release was measured in cells grown to confluence (Day 0, filled circles) or in cells stratified by the addition of FBS and calcium for 3 days (Day 3, open circles). Data shown are means ± SEM for triplicate experiments using cells isolated from 4 normal and 4 IC/PBS patients. **p<0.01,when compared to unstimulated controls.

To determine whether the loss of PGE_2_ release was consistent in IC/PBS urothelial cells following differentiation into stratified cultures, we measured PGE_2_ release in tryptase-stimulated human urothelial cells (HUC) and immortalized urothelial cells from normal and IC/PBS patients after 0 and 3 days of Ca/FBS addition. PGE_2_ release from confluent monolayers of HUC or immortalized urothelial cells isolated from normal subjects was significantly increased following tryptase stimulation and remained increased over the 60 min stimulation period studied (Fig 2, upper and middle panels, filled circles). Following 3 days in culture with added Ca/FBS, HUC and immortalized urothelial cells from normal bladders demonstrated a greater increase in PGE_2_ release in response to tryptase stimulation at each time point measured (Fig 2, upper and middle panels, open circles). In contrast, immortalized urothelial cells isolated from IC/PBS patients demonstrated no significant increase in PGE_2_ release in the presence of tryptase at either day 0 or day 3 of Ca/FBS addition (Fig 2, lower panel).

**Fig 3:**
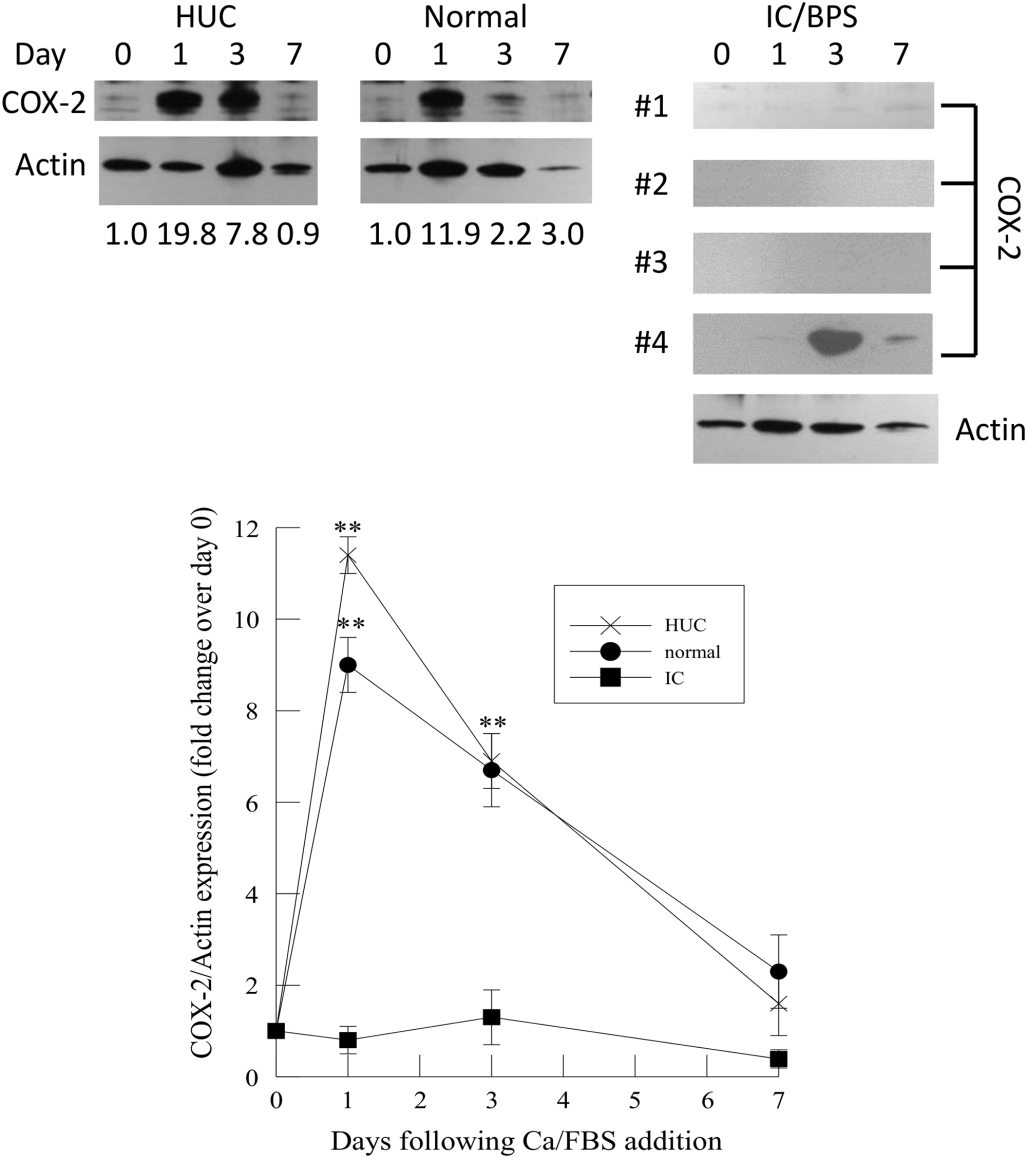
Expression of cyclooxygenase-2 (COX-2) in human urothelial cells, and immortalized urothelial cells from normal and IC/PBS patients after Ca/FBS addition. Upper panel: Representative immunoblots of COX-2 and actin expression in human urothelial cells (HUC) and immortalized urothelial cells from normal and IC/PBS patients. Lower panel: COX-2 immunoprotein was compared to actin for each sample and normalized to 1 for samples at Day 0. COX-2 immunoprotein changes were analyzed in three separate cells cultures of human urothelial cells (HUC, n = 3) or immortalized urothelial cells from normal (n = 4) or IC/PBS patients (n = 4, denoted in figure as #1–4). Data shown are mean ± SEM. ** p<0.01 when compared to day 0.

We measured expression of synthesis and degradation enzymes for PGE_2_ in urothelial cells during differentiation. Immunoblot analysis of HUC and immortalized urothelial cells isolated from normal patients demonstrated a significant increase in COX-2 expression after 1 and 3 days of Ca/FBS addition (Fig 3, lower panel). COX-2 expression had returned to control levels by day 7 (Fig 3, lower panel). Surprisingly, COX-2 immunoprotein was only detectable in urothelial cells obtained from 1 of 4 IC/PBS patients, under similar experimental conditions (Fig 3, lower panel). These data suggest that little, if any, PGH_2_ may be produced in IC/PBS urothelial cells at any time during differentiation.

**Fig 4:**
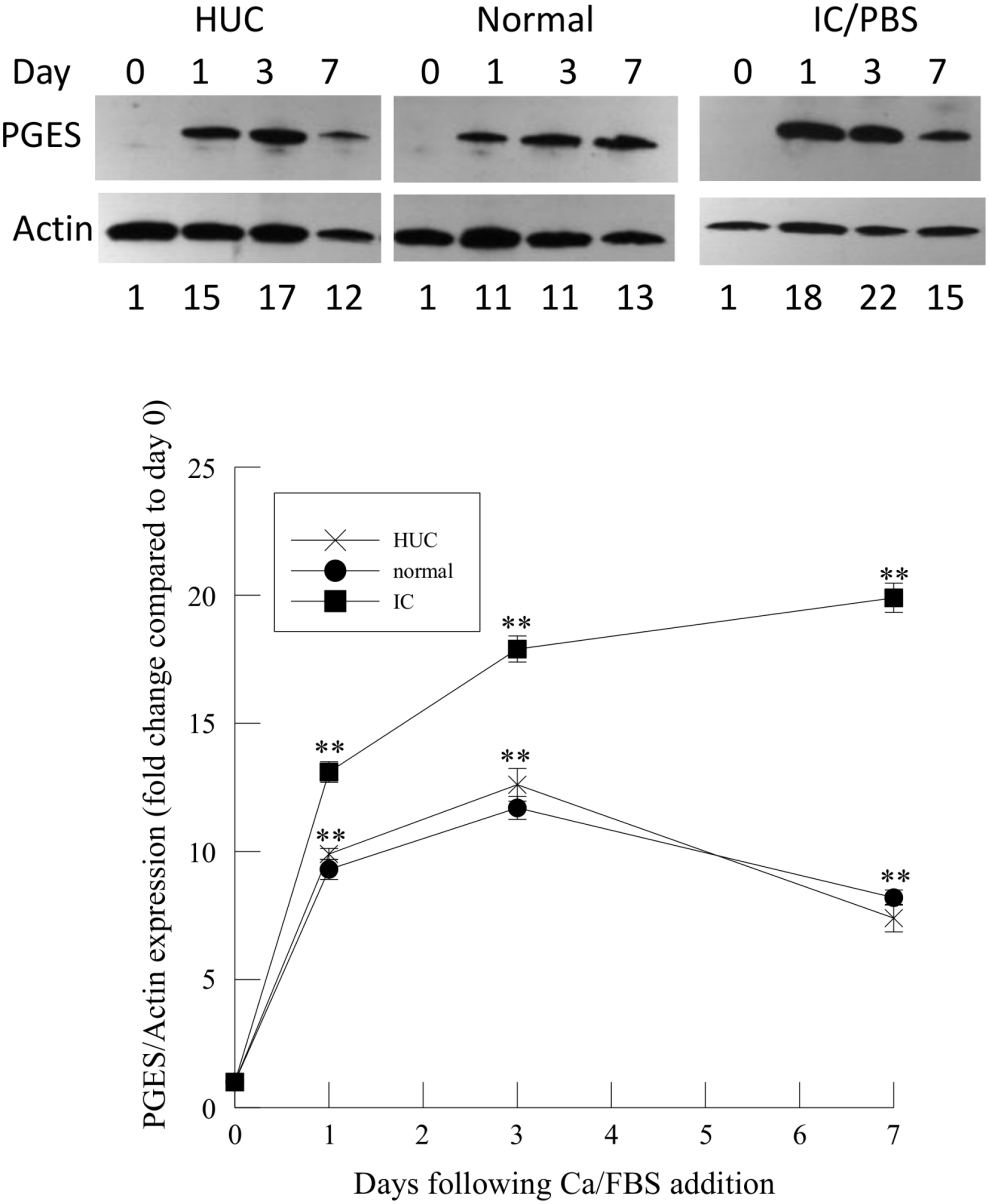
Expression of prostaglandin E synthase (PGES) in human urothelial cells, and immortalized urothelial cells from normal and IC/PBS patients after Ca/FBS addition. Upper panel: Representative immunoblots of PGES and actin expression in human urothelial cells (HUC) and immortalized urothelial cells from normal and IC/PBS patients. Lower panel: PGES immunoprotein was compared to actin for each sample and normalized to 1 for samples at Day 0. PGES immunoprotein changes were analyzed in three separate cells cultures of human urothelial cells (HUC, n = 3) or immortalized urothelial cells from normal (n = 4) or IC/PBS patients (n = 4). Data shown are mean ± SEM. ** p<0.01 when compared to day 0.

Immunoblot analysis of HUC or immortalized urothelial cells from normal and IC/PBS patients demonstrates a significant increase in PGES expression following Ca/FBS stimulation (Fig 4, lower panel). Increased PGES expression in immortalized urothelial cells from IC/ PBS patients was greater than in HUC and immortalized urothelial cells from normal subjects following the addition of Ca/FBS (Fig 4, lower panel). This increased expression may be a result of the absence of COX-2 expression in IC/PBS urothelial cells (Fig 3).

**Fig 5:**
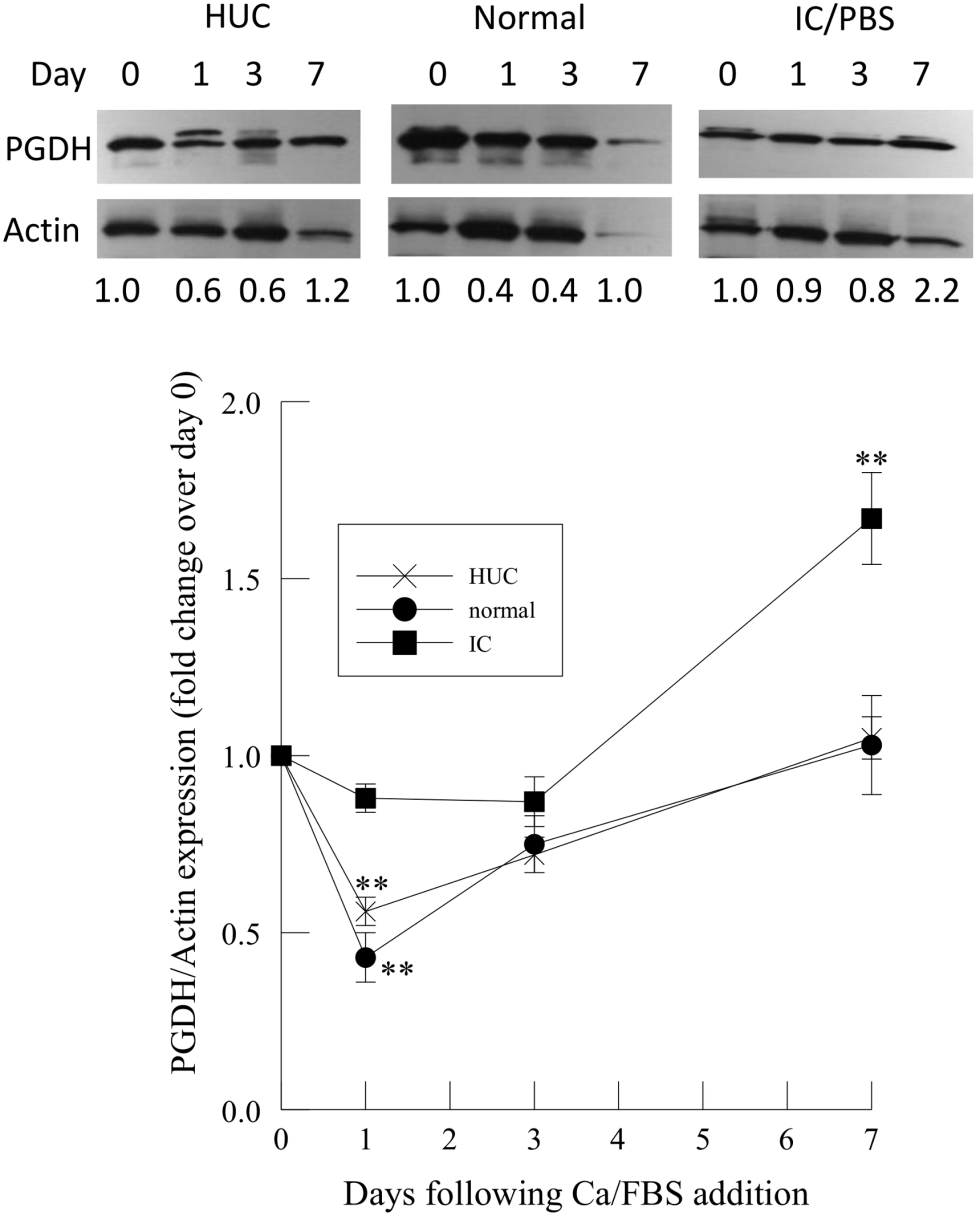
Expression of 15-hydroxyprostaglandin dehydrogenase (PGDH) in human urothelial cells (HUC) and immortalized urothelial cells from normal and IC/PBS patients after Ca/FBS addition. Upper panel: Representative immunoblots of PGES and actin expression in human urothelial cells (HUC) and immortalized urothelial cells from normal and IC/PBS patients. Lower panel: PGES immunoprotein was compared to actin for each sample and normalized to 1 for samples at Day 0. PGES immunoprotein changes were analyzed in three separate cells cultures of human urothelial cells (HUC, n = 3) or immortalized urothelial cells from normal (n = 4) or IC/PBS patients (n = 4). Data shown are mean ± SEM. ** p<0.01 when compared to day 0.

15-hydroxyprostaglandin dehydrogenase (PGDH) expression was significantly decreased in HUC and immortalized urothelial cells from normal bladders after 1 day of Ca/FBS addition, but had returned to control levels by day 7 of differentiation (Fig 5, lower panel). In contrast, PGDH expression in immortalized urothelial cells isolated from IC/PBS bladders was not significantly changed after 1 and 3 days after Ca/FBS addition, but was increased approximately 2-fold over control at 7 days (Fig 5, lower panel).

Taken together, the data in this study suggest that the absence of PGE_2_ release in IC/PBS urothelial cells is due to both decreased synthesis and increased degradation during differentiation.

## Discussion

IC/PBS is a debilitating disease associated with voiding dysfunction and pain. The pathogenesis of IC/PBS is likely multifactorial, with proposed etiologies including bladder urothelial cell impairment, mast cell involvement and inhibition of urothelial cell growth with antiproliferative factor (APF) [18–20]. Our previous study demonstrated a failure to produce PGE_2_ in monolayers of tryptase-stimulated immortalized urothelial cells isolated from IC/PBS bladders [10]. The present study now demonstrates that the absence of PGE_2_ release occurs in immortalized urothelial cells from IC/PBS bladders following differentiation. IC/PBS is associated with epithelial sloughing, thinning and denudation [21]. Following injury, urothelial cell regeneration involves spreading and migration of cells to re-epithelialize the wounded area. Prostaglandins favor cell migration and wound healing in a variety of tissues, including corneal, intestinal and airway epithelium [22–24]. Our finding that IC/PBS cells fail to release PGE_2_ in response to tryptase may represent a mechanism whereby the urothelium is vulnerable in settings where there is mast cell activation such as the IC/PBS bladder.

The urothelial cell lining of the bladder has a low constitutive turnover rate, but an exceptionally high capacity for regeneration under normal conditions. In *in vitro* studies, normal human urothelial cells grown under serum-free and low calcium conditions display a highly proliferative phenotype with little or no expression of urothelial differentiation markers. In the presence of serum and increased calcium, cells form a stratified, functionally-differentiated urothelium (Fig 1). Urothelial cell differentiation is associated with alterations in cell signaling. Urothelial cells in the IC/PBS bladder may also follow an aberrant differentiation program leading to altered synthesis of several proteins involved in barrier function. Our data suggest that the lack of PGE_2_ release from IC/PBS immortalized urothelial cells is preserved during differentiation.

PGE_2_ production is initiated by activation of PLA_2_, which releases arachidonic acid from the *sn*-2 position of membrane phospholipids. Activation of PLA_2_ to release arachidonic acid and COX, which converts arachidonic acid to the intermediate prostaglandin precursor PGH2, represent the two crucial rate limiting steps for the prostaglandin biosynthetic pathway. It is proposed that production of prostaglandins within minutes is mediated by constitutively active COX-1 and more delayed prostaglandin production by inducible COX-2 [11, 25]. COX-1 immunoprotein has been shown to be located in the basal urothelium, lamina propria and the surface of the inner muscle bundles in the bladder, with COX-2 immunoprotein associated with the cell nuclei. Acetic acid-induced bladder irritation was attenuated when both COX-1 and COX-2 were inhibited pharmacologically, but not affected when COX isoforms were inhibited selectively, suggesting the prostaglandin production in the bladder is mediated via both isoforms [26]. Changes in bladder COX-2 have been implicated in hemorrhagic cystitis, E. coli infection and bladder cancer [27–29]. Immunoblot analysis of COX-1 expression did not detect any significant changes during differentiation of any cell type used in these studies (data not shown). Our data suggest that PGE_2_ production in response to tryptase is mediated via COX-2 activity, but we cannot rule out a role for COX-1.

Our previous data in undifferentiated urothelial cells suggested that the lack of PGE_2_ production in IC/PBS urothelium was a direct result of decreased synthesis via COX-2 and/or PGES and increased metabolism by PGDH. The data herein indicate that there are significant alterations in these enzymes during differentiation and that there are major differences in immunoprotein expression between urothelial cells isolated from normal and IC/PBS bladders. HUC and immortalized urothelial cells isolated from normal bladders demonstrate a significant increase in COX-2 and PGES immunoprotein at 1 and 3 days post addition of Ca/FBS. These changes were accompanied by an increase in PGE_2_ release in unstimulated urothelial cells and in response to tryptase at day 3 when compared to the release observed at Day 0. Since PGE_2_ is implicated in cell migration and wound healing, the increases in COX-2 and PGES at days 1 and 3 of Ca/FBS may facilitate rapid cell growth and differentiation at these time points. The absence of PGE_2_ in IC/PBS bladder urothelial cells may contribute to the impaired wound healing and urothelial repair observed in this disease. A particularly striking difference in our studies was that we were not able to detect COX-2 immunoprotein in 3 of the 4 IC/PBS isolations.

## Conclusion

Our study suggests that alterations in COX-2, PGES or PGDH expression and the resultant loss of PGE_2_ production in IC/PBS could further contribute to loss of protection and wound repair of the urothelium. This may represent a key impairment in the normal protection and repair of the urothelium during inflammatory events in the progression of this disease.
